# Comorbidity and outcomes among hospitalized patients with stroke: a nationwide inpatient analysis

**DOI:** 10.3389/fneur.2023.1217404

**Published:** 2023-10-17

**Authors:** Wei Chen, Dong Li

**Affiliations:** ^1^Department of Neurosurgery, West China Hospital of Sichuan University, Chengdu, China; ^2^West China Biomedical Big Data Center, West China Hospital of Sichuan University, Chengdu, China; ^3^Med-X Center for Informatics, Sichuan University, Chengdu, China; ^4^Department of Emergency Medicine, Harbor-UCLA Medical Center, Torrance, CA, United States

**Keywords:** comorbidities, stroke, older people, prevalence, healthcare burden

## Abstract

**Objective:**

We aimed to characterize healthcare utilization and comorbidity outcomes among hospitalized elderly stroke patients using a nationally representative dataset in the United States.

**Methods:**

Using the 2019 National Inpatient Sample, patients aged 65 years or older with and without comorbidities who were hospitalized for acute stroke were identified using the International Classification of Diseases, Tenth Revision, Clinical Modification codes. Patient comorbidities were identified with the use of the Elixhauser Comorbidity Index. The prevalence of comorbidities, in-hospital mortality, length of stay, and total hospital costs were analyzed for these patients.

**Results:**

Within 451,945 patients (mean age 78 years, 54.1% women, 73.7% white), we observed that more than 90% of patients had a minimum of two comorbidities. The median number of comorbidities was 4.0 (IQR 3.0–6.0). There was significant variation in the prevalence rate of comorbidities. The five most common comorbidities were uncomplicated hypertension (55.4%), paralysis (40.1%), congestive heart failure (39.8%), various neurological illnesses (38.3%), and complex hypertension (32.5%). After adjusting for patient- and hospital-level characteristics, a statistically significant association was observed between comorbidities and various adverse outcomes. Specifically, comorbidities were found to be significantly associated with an increased risk of inpatient mortality (odds ratio: 1.09; 95% CI: 1.08–1.11; *P* < 0.001), a longer duration of hospitalization (0.68 days; 95% CI: 0.66–0.71; *P* < 0.001), and higher total cost ($1,874.9; 95% CI: 1,774.6–1,975.2; *P* < 0.001).

**Conclusion:**

This national data suggests that comorbidity is common among hospitalized older stroke patients and substantially increases the healthcare burden and inpatient mortality in the United States. These findings underscore the integration of comorbidity management into the care of older stroke patients.

## 1. Introduction

Comorbidities, in the context of medical research, refer to the simultaneous presence of two or more diseases in a patient, particularly in relation to an index disease under investigation (1). As the global population experiences growth and aging, coupled with elevated risk factors for non-communicable diseases, there has been an anticipated rise in the prevalence of comorbidities (2, 3). Stroke, a non-communicable ailment, has significant long-term implications on health and quality of life, and its prevalence is notably higher in older individuals. Consequently, the incidence of comorbidities among stroke patients is prevalent (4–7). This growing prevalence underscores the increasing academic and clinical interest in understanding the patterns of comorbidity or multimorbidity (the coexistence of multiple diseases in a single individual) in older patients hospitalized due to stroke. Such interest stems from the challenges comorbidities introduce in clinical settings, such as the complexity they add to treatment strategies. While numerous studies have delved into the implications of comorbidities in stroke (7–9), there remains a paucity of information on the most recent prevalence and healthcare burden among elderly stroke patients on a national scale. A comprehensive understanding of the prevalence of comorbidities and their impact on healthcare resources is pivotal for informed public health policymaking and resource distribution for the care of older stroke patients.

To bridge these knowledge gaps, our study utilizes a nationally representative database, encompassing the most extensive collection of elderly stroke cases to date. Our objective is to assess the current prevalence, healthcare engagement, and outcomes of comorbidities in adults aged 65 years or older who are hospitalized due to acute stroke.

## 2. Methods

### 2.1. Study design and data source

A case–control (patients with and without comorbidities exposure) retrospective cohort study design was performed utilizing the National Inpatient Sample (NIS), the largest publicly available all-payer inpatient healthcare database, which intends to produce regional and national estimates of inpatient healthcare utilization, charges, quality, and outcomes in the United States. The NIS comprised discharge data from a sample covering more than 95% of the US population, estimating more than 35 million hospitalizations nationally. Starting in 2012, the NIS captured a 20% stratified sample of discharges from all U.S. community hospitals. Further details on the NIS design are available through HCUP's online resources (http://www.hcup-us.ahrq.gov). For the proposed study, all patients in this study were hospitalized in 2019. This study received an informed consent waiver from the institutional review board of West China Hospital, Sichuan University because it involved no more than minimal risk to subjects and used open-source de-identified medical records for secondary data analysis.

### 2.2. Specific study population

We identified patients aged 65 years or older who were hospitalized with a primary diagnosis of stroke during the data extraction period. This identification was based on the International Classification of Diseases, Tenth Revision, Clinical Modification (ICD-10-CM) codes. These codes encompass hemorrhagic stroke (subarachnoid hemorrhage [I60.xx] and intracerebral hemorrhage [I61.xx]), ischemic stroke (I63.xx, I64.xx, and H34.1), and transient ischemic attack (TIA) (G45.xx), as delineated in prior studies. (10, 11). Our study focused on patients admitted in 2019 to ensure a contemporary cohort representation. We excluded elective hospitalizations to specifically target acute stroke cases and omitted patients with incomplete demographic data.

### 2.3. Patient-and hospital-level characteristics

Utilizing the NIS data, we discerned both patient-level and hospital-specific characteristics. Patient attributes comprised age, sex, racial background (categorized as white, black, Hispanic, Asian and Pacific Islander, Native American, and others), primary payment method (options being Medicare, Medicaid, private, self-pay, no charge, and others), and income percentile based on residential zip code. Comorbidities were identified using the HCUP Clinical Classification Software definitions, and their assessment was facilitated by the Elixhauser Comorbidity Index (ECI), which encompasses 31 comorbidity markers. (12, 13). Hospital-related data included bed capacity (small, medium, or large), educational status (rural, urban non-teaching, or urban teaching), and geographical location (northeast, midwest, south, or west). In our study, most variables had minimal missing data (<0.5%), with race (2.3%) and median household income (1.5%) being the exceptions.

### 2.4. Study outcomes

Our analysis focused on outcomes such as in-hospital mortality, hospital stay duration, total hospitalization expenses, and discharge destination (routine discharge or otherwise). Since the total billed amount does not accurately reflect the cost of hospital services, we employed a charge-to-cost conversion ratio to determine the actual cost borne by the payer.

### 2.5. Statistical analysis

All statistical evaluations were conducted using the sampling frame and weights provided by the NIS to derive national estimates. Hospitalization descriptive statistics were presented in terms of mean (standard deviation) and median (interquartile range) for continuous variables and frequencies (percentages) for categorical ones. We probed the relationships between comorbidities and various outcomes such as in-hospital mortality, length of stay, hospitalization cost, and routine discharge. This was achieved using multivariable linear or logistic regression models, presented as odds ratios (ORs) or β values with their respective 95% confidence intervals (CIs). Given the mortality rate difference between hemorrhagic and ischemic strokes, we conducted a subgroup analysis. Our multivariable regression models accounted for factors such as age, sex, race, income, primary payer, hospital size, teaching status, and region. A two-tailed *P*-value below 0.01 was deemed to be statistically significant. All computations were executed using Stata 17 (StataCorp LLC, College Station, Texas).

## 3. Results

Our conclusive study sample encompassed 90,389 hospitalizations with primary discharge diagnoses of stroke in patients aged 65 years or older in the U.S. This translates to an estimated 451,945 stroke hospitalizations nationally after applying sampling weights. The average age of our study cohort was 78.0 years, with a standard deviation of 0.04, 54.1% were female patients, and 73.7% identified as white. Table 1 provides a detailed breakdown of the study population's characteristics.

**Table 1:** Patient- and hospital-level characteristics of the study population.

**Characteristics**	**Overall**
Total no. of weighted hospitalizations	451,945
**Elixhauser comorbidity index**	
Mean (SD)	4.3 (0.01)
Median (IQR)	4.0 (3.0–6.0)
**Age in years**	
Mean (SD)	78.0 (0.04)
Median (IQR)	78.0 (71.0-85.0)
Female, *N* (%)	244,280 (54.1)
**Race/ethnicity**, ***N*** **(%)**	
White	333,210 (73.7)
Black	59,030 (13.1)
Hispanic	31,915 (7.1)
Asian and Pacific Islander	14,990 (3.3)
Native American	1,750 (0.4)
Others	11,050 (2.4)
**Median household income**, ***N*** **(%)**	
First QT	124,965 (27.7)
Second QT	115,315 (25.5)
Third QT	114,210 (25.3)
Fourth QT	97,455 (21.6)
**Primary expected payer**, ***N*** **(%)**	
Medicare	401,515 (88.8)
Medicaid	6,740 (1.5)
Private insurance	33,110 (7.3)
Self-pay	3,555 (0.8)
No charge	210 (0.0)
Other	6,815 (1.5)
**Hospital bed size**, ***N*** **(%)**	
Small	83,085 (18.4)
Medium	132,010 (29.2)
Large	236,850 (52.4)
**Hospital teaching status**, ***N*** **(%)**	
Rural	31,310 (6.9)
Urban non-teaching	78,670 (17.4)
Urban teaching	341,965 (75.7)
**Hospital region**, ***N*** **(%)**	
Northeast	84,865 (18.8)
Midwest	96,085 (21.3)
South	184,2420 (40.8)
West	86,575 (19.2)
In-hospital mortality, *N* (%)	26,350 (5.8)
**Length of stay (days)**	
Mean (SD)	4.8 (0.04)
Median (IQR)	3.0 (2.0-6.0)
**Hospital cost ($)**	
Mean (SD)	15,809.1 (188.3)
Median (IQR)	10,022.1 (6,695.5–17,368.9)

Frequencies (%) in the columns may not sum to 100% due to rounding. Data weighted using sampling weights to achieve nationally representative estimates. IQR, interquartile rang; SD, standard deviation.

**Figure 1:**
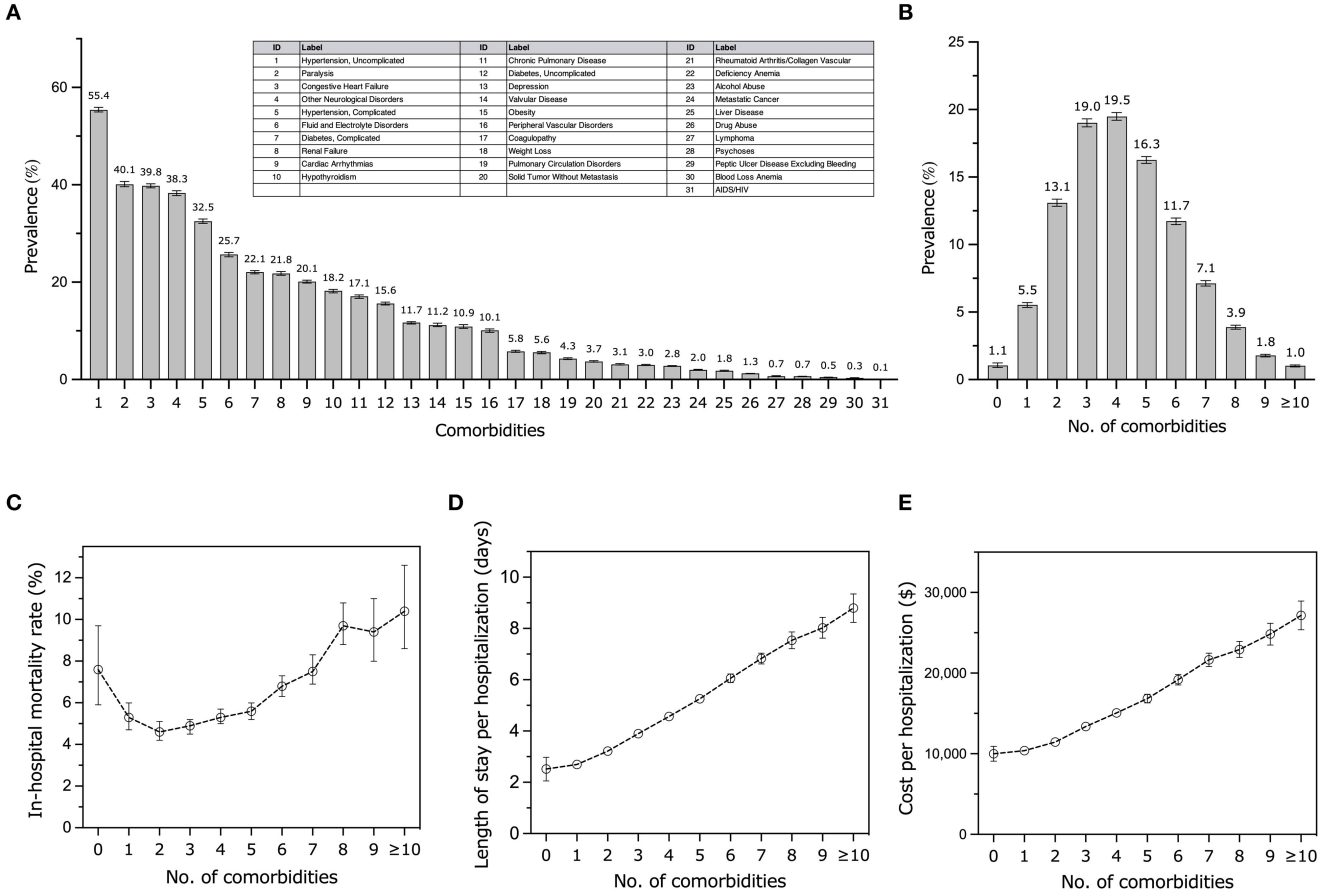
Prevalence and outcomes of comorbidities among elderly patients hospitalized with acute stroke. **(A)** Prevalence of each comorbid condition. Error bars illustrate a 95% confidence interval (CI). **(B)** Distribution of comorbidity count. Error bars represent 95% CI. **(C)** Estimated inpatient mortality rate by number of comorbidities and the adjusted odds ratio based on multivariable logistic regression. **(D, E)** Estimated mean length of stay per hospitalization and estimated median cost by number of comorbidities and the adjusted β based on multivariable linear regressions.

Figure 1A illustrates the varying prevalence rates of comorbidities, ranging between 0.1% and 55.4%. The top five comorbidities, in terms of prevalence, were hypertension (uncomplicated) at 55.4%, paralysis at 40.1%, congestive heart failure at 39.8%, other neurological disorders at 38.3%, and complicated hypertension at 32.5%. In our sample, over 90% of patients had a minimum of two comorbidities, as shown in Figure 1B. Apart from the patients with no (1.1%) or only one (5.5%) comorbidity, the remainder had at least two or more comorbidities (93.4%). The median number of comorbidities stood at 4.0, with the majority having either three or four comorbidities (19.0% and 19.5%, respectively). Overall, a rise in the number of comorbidities correlated with increased healthcare burdens and outcomes in elderly stroke patients. After adjusting for various factors, comorbidities were linked with elevated inpatient mortality rates (OR: 1.09; 95% CI: 1.08–1.11; *P* < 0.001), longer hospital stays (0.68 days; 95% CI: 0.66–0.71; *P* < 0.001), increased costs ($1,874.9; 95% CI: 1,774.6–1,975.2; *P* < 0.001), and reduced routine discharges (OR: 0.75; 95% CI: 0.74–0.76; *P* < 0.001), as depicted in Figures 1C–E and the Supplementary Tables.

## 4. Discussion

Utilizing the comprehensive NIS, the largest hospitalization database in the United States, we present a detailed profile of comorbidities among elderly stroke patients. Our analysis underscores the prevalent nature of comorbidities in hospitalized older stroke patients, emphasizing their significant contribution to the healthcare burden and associated adverse outcomes. To the best of our knowledge, this represents the inaugural study that delineates comorbidities among elderly stroke admissions using the NIS, offering a contemporary national viewpoint on the prevalence and healthcare implications of these comorbidities.

Our results resonate with prior studies that have highlighted the substantial prevalence and healthcare implications of comorbidities in stroke patients (6, 7, 9, 14, 15). These findings accentuate the imperative of assimilating comorbidity management within stroke care protocols. This study augments our comprehension by presenting current national estimates of the burden of comorbidities, with a particular emphasis on older stroke patients. As the geriatric demographic in the US expands, it heralds profound shifts and challenges in the realms of both comorbidities and stroke care. A nuanced grasp of the epidemiology, healthcare implications, and outcomes of these comorbidities is pivotal for informed clinical decision-making, especially for older stroke patients with concurrent conditions. This knowledge is instrumental in enhancing care quality and outcomes for this burgeoning demographic. Our insights underscore the pressing need for strategic interventions to address comorbidities in elderly stroke patients.

The robust sample size of our study offers a realistic snapshot of the current national prevalence of these comorbidities in stroke, making our findings broadly applicable across the United States. Nevertheless, it is essential to interpret our results in light of certain limitations. The NIS, being an administrative database, relies on ICD-10-CM codes and may be prone to coding inaccuracies, such as miscoding and under-coding.

Our study predominantly utilized the ECI, comprising 31 comorbidities. However, careful interpretation is warranted, especially concerning conditions such as “paralysis” and “other neurological disorders,” which may be more aptly associated with stroke diagnoses rather than as separate comorbidities (16). Additionally, conditions such as “alcohol abuse” might be more fittingly categorized as stroke risk factors, while “fluid and electrolyte disorders” might be more aptly viewed as complications. Future endeavors should delve into the intricate interplay and management strategies, adopting a multidisciplinary approach, especially when addressing comorbidities and stroke in the aging population.

## Data availability statement

The data analyzed in this study was obtained from the Agency for Healthcare Research and Quality (AHRQ), Healthcare Cost and Utilization Project (HCUP; https://hcup-us.ahrq.gov/), National Inpatient Sample (NIS), the following licenses/restrictions apply: Users must complete HCUP data use training, sign and submit a Data Use Agreement before accessing HCUP datasets. Requests to access these datasets should be directed to HCUP, hcup@ahrq.gov.

## Ethics statement

Ethical review and approval was not required for the study on human participants in accordance with the local legislation and institutional requirements. Written informed consent from the patients/participants or patients/participants' legal guardian/next of kin was not required to participate in this study in accordance with the national legislation and the institutional requirements.

## Author contributions

Conceptualization was led, methodology was developed, and the manuscript's original draft was penned by WC and DL. Figures were prepared and funding was secured by WC. All authors reviewed and approved the manuscript.
