# Supplementary material for: Comorbidity and outcomes among hospitalized patients with stroke: a nationwide inpatient analysis
Source: Front Neurol. 2023 Oct 17;14:1217404. doi: 10.3389/fneur.2023.1217404 (PMC10616246; doi:10.3389/fneur.2023.1217404)
Supplement: Supplementary file 1 [file Data_Sheet_1.DOCX]

**Table S1 In-hospital mortality stratified by number of comorbidities and type of stroke.**

| **Overall** | | | |
| --- | --- | --- | --- |
| No. of comorbidities | In-hospital mortality | Lower 95% CI | Upper 95% CI |
| 0 | 7.6% | 5.9% | 9.7% |
| 1 | 5.3% | 4.7% | 6.0% |
| 2 | 4.6% | 4.2% | 5.1% |
| 3 | 4.9% | 4.5% | 5.2% |
| 4 | 5.3% | 5.0% | 5.7% |
| 5 | 5.6% | 5.2% | 6.0% |
| 6 | 6.8% | 6.3% | 7.3% |
| 7 | 7.5% | 6.9% | 8.3% |
| 8 | 9.7% | 8.8% | 10.8% |
| 9 | 9.4% | 8.0% | 11.0% |
| >=10 | 10.4% | 8.6% | 12.6% |
| **Acute hemorrhagic stroke** | | | |
| No. of comorbidities | In-hospital mortality | Lower 95% CI | Upper 95% CI |
| 0 | 32.0% | 25.0% | 39.8% |
| 1 | 29.3% | 26.0% | 32.9% |
| 2 | 26.3% | 24.0% | 28.8% |
| 3 | 24.6% | 22.7% | 26.6% |
| 4 | 21.4% | 19.6% | 23.4% |
| 5 | 19.5% | 17.7% | 21.6% |
| 6 | 20.7% | 18.5% | 23.2% |
| 7 | 19.7% | 16.9% | 22.8% |
| 8 | 20.3% | 16.3% | 25.0% |
| 9 | 15.0% | 10.5% | 20.9% |
| >=10 | 13.8% | 8.4% | 21.9% |
| **Acute ischemic stroke** | | | |
| No. of comorbidities | In-hospital mortality | Lower 95% CI | Upper 95% CI |
| 0 | 2.3% | 1.2% | 4.4% |
| 1 | 1.6% | 1.2% | 2.0% |
| 2 | 1.6% | 1.3% | 1.8% |
| 3 | 2.2% | 1.9% | 2.4% |
| 4 | 3.3% | 3.0% | 3.6% |
| 5 | 3.8% | 3.5% | 4.2% |
| 6 | 5.1% | 4.6% | 5.5% |
| 7 | 6.0% | 5.4% | 6.7% |
| 8 | 8.6% | 7.6% | 9.7% |
| 9 | 8.8% | 7.4% | 10.4% |
| >=10 | 10.0% | 8.2% | 12.2% |

**Table S2 Rate of routine discharge stratified by number of comorbidities.**

| **No. of comorbidities** | **Rate of routine discharge** | **Lower 95% CI** | **Upper 95% CI** |
| --- | --- | --- | --- |
| 0 | 58.6% | 53.2% | 63.8% |
| 1 | 56.2% | 54.7% | 57.7% |
| 2 | 45.3% | 44.2% | 46.3% |
| 3 | 35.7% | 34.9% | 36.5% |
| 4 | 29.7% | 29.0% | 30.5% |
| 5 | 24.0% | 23.3% | 24.7% |
| 6 | 19.4% | 18.6% | 20.2% |
| 7 | 16.0% | 15.1% | 16.9% |
| 8 | 14.1% | 12.9% | 15.3% |
| 9 | 11.1% | 9.7% | 12.8% |
| >=10 | 9.2% | 7.5% | 11.3% |
